# Factors Affecting Gut Microbiome in Daily Diet

**DOI:** 10.3389/fnut.2021.644138

**Published:** 2021-05-10

**Authors:** Qi Su, Qin Liu

**Affiliations:** Department of Medicine and Therapeutics, The Chinese University of Hong Kong, Hong Kong, China

**Keywords:** additives, cooking, diet, food ingredients, gut microbiome, processing

## Abstract

There is a growing recognition that a good diet can help people maintain mental and physical health, while a bad one will cause the disorder of body function, and even lead to several diseases. A lot of attentions have been devoted to analyze every possible health-related factor in the daily diet, including food ingredients, additives, and cooking process. With the support of high-throughput sequencing technology, there is accumulating evidence gradually clarifying that most of these factors are mainly through the interactions with gut microbiome to trigger downstream effects. The gut microbiome may be able to act as a very sensitive mirror in response to human daily diet. A complex network of interactions among diet, gut microbiome, and health has been gradually depicted, but it is rarely discussed from a more comprehensive perspective. To this end, this review summarized the latest updates in diet-gut microbiome interactions, analyzed most identified factors involved in this process, showed the possibility of maintaining health or alleviating diseases by diet intervention, aiming to help people choose a suitable recipe more accurately.

## Introduction

Food provides energy and nutrition that has a great impact on health, mainly by changing the gut conditions and then influencing host immunology homeostasis. Trillions of microbes settle in the gut and play fundamental roles in metabolism, endocrine, neuronal, immune and many other aspects of body function. Notably, diet can greatly influence gut microbiome (1), which can greatly impact host's health. Given the inextricable relationship among diet, gut microbiome, and health, numerous studies have been carried out to explore the underlying mechanism (2). Especially at present, obesity, inflammatory bowel disease, allergic diseases, cognitive aging, Alzheimer's disease, and many other non-communicable diseases have become an important health problem in developed and developing countries (3–5), highlighting a need to understand how to attenuate these problems, while diet intervention seems to be the most effective and pleasant way (6, 7).

Dietary habits have a profound effect in shaping the gut microbiome in real time since birth. Microbes colonize the gut immediately after birth, and such early development of gut microbiome is thought to be driven and regulated, at least in part, by specific compounds present in breast milk (8, 9). Several host and external factors modulate the establishment of the immunity during the fetal and early postnatal life, however, few are as important as the interaction with commensal microbes, which is not only the most intimate environmental exposure but also represents a challenge to the development of host (10–13). For example, infants with lower relative abundance of *Bifidobacterium, Akkermansia*, and *Faecalibacterium* are at higher risk of CD4+ T cell dysfunction, which might induce to childhood atopy and asthma (10), and the dysbiosis of these microbes is currently clearly related to unsuitable formula feeding (12, 13), showing the importance of early feeding of babies. On the other hand, some *Bifidobacterial* species have evolved to utilize glycan in human secretion, which represents the adaptive ability at transformation of symbiotic microorganisms to the host, and it is believed that both sides will benefit (14–16) *via* microbiome-host cell interactions.

Infancy is the starting point for the development of gut microbiome, which will gradually mature in the next 3 years and undergo changes over a life-long time (17). From infants to adults, the host itself and gut microbes are co-evolving and interacting, while it is important to provide a harmonious environment for each side (18–20). In healthy adults, the overall composition of the gut microbiome can be stable for several years (21), but the relative abundance of each member contained in is highly variable (22). Long term dietary habits affect the development and maturation of gut microbiome, but this does not mean that temporary adjustment is not possible to achieve the desired aims, especially foodborne microorganisms, including bacteria, fungi and even viruses, can quickly colonize the gut. A study of human diet intervention showed that changes or adjustments of food types in an extremely short time can quickly change the structure of gut microbiome, which will become completely different in 3 or 4 days, and overwhelms the individual differences in microbial gene expression and personal genetic background (22, 23), showing the universality and potential of the dietary intervention (23). Therefore, no matter for any stage of life, choosing a reasonable diet has an obvious salutary influence on health.

In the long history, people's eating habits are also gradually changing. From the original menu shared by primitive humans and wild animals, such as orangutans and monkeys, to the farming period when people ate certain kinds of grains with little meat, and then to the modern food produced by complex process and containing a variety of additives in the industrial era, gut microbiome itself has also undergone the challenges of these changes, and become completely different. It's still hard to say whether this change is good or bad. The previous study concerning the traditional populations gives us an overview of human-related microbes influenced by industrialization, and also a window into the co-evolution between the microbiome and human, showing that not only the microbiome from the Hadza hunter-gatherers of Tanzania seasonally shift in bacterial taxa, diversity, and carbohydrate utilization, but also shares certain microbes with several other traditional populations that are almost rare or absent from microbiomes of modern countries (24, 25). Given the positive correlation between the diversity of gut microbiome and health, there is no doubt that the transition from the traditional farming era to the industrial age has resulted in the loss of critical organisms and functionality for industrialized populations, even if their effects on health have not been elucidated. There is no doubt that food in the industrial era has not made our gut microbiome more resilient, which may be the reason why the incidence rate of many non-communicable diseases is increasing with the development of society and is more significant in developed countries (3, 26–28). As a result, for most people with long-term unchanged recipes, their gut microbial diversity is gradually lost, which in turn affects the host, causing more diseases. For example, the western diet (ultra-processed foods with excess fat, sugar, additives, and a very small amount of micronutrients and dietary fiber) is closely related with the current prevalence of obesity and several other metabolic diseases, because the environment shaped in the gut by this dietary patterns provides fertile soil for microbes that can induce diverse inflammatory diseases (29–31). Therefore, clarifying the role of the microbiome in diet-related diseases is of great importance to precision medicine, dietary recommendation, and food production practice.

At present, it has been widely recognized that there are a large amount of microbial species that settle in the gut, while the composition and function of it are closely related to the diseases of the digestive, nervous, respiratory, metabolic, and cardiovascular system (32). A lot of work has focused on exploring the potential relationship and related mechanisms of microbial mediated diseases, and many new findings have been used to guide patients to choose appropriate long-term or short-term diet intervention to alleviate the disease or accelerate the recovery (33, 34). Most of them demonstrated that low energy or high fiber diet is very helpful to revitalize gut microbiome, which happens to provide an improvement suggestion for those who have already adapted to the western diet, especially for people who are suffering from obesity or autism (35–39). With the help of advanced sequencing technology and analysis technology, we can accurately depict the profile of gut microbiome in people with or without a certain condition, but it is difficult to say whether these differences are the cause or the consequences of related diseases. Relatively few randomized, clinically controlled dietary interventions targeting gut microbiome have been reported in humans, thus more accurate experiments should be carried out as soon as possible to make up for these gaps.

Now, the accumulated evidence is gradually clarifying the relationship between diet, microbiome and disease. This review will analyze the effects of diet on the gut microbiome from different aspects, including food (carbohydrates, fats, proteins, minerals, and vitamins), additives and different cooking and processing etc., and try to establish a complete framework from these perspectives, show the focus of related research, and lay a foundation for future research and development.

## Carbohydrates

Carbohydrates are composed of carbon, hydrogen, and oxygen, which are the most abundant organic compounds with broad-spectrum chemical structure and biological functions (40). They can be expressed by the general formula C_x_(H_2_O)_y_ and works as the main source of energy for the human body. Generally, there are two types of carbohydrates in food: the effective carbohydrates that people can totally digest and then absorb as well as the ineffective counterparts that people can't digest by themselves, namely non-digestible carbohydrates, which are far away from the way digestible carbohydrates go in the gut. Non-digestible carbohydrates get through the first part of the digestive tract completely to the ileum and colon, and then will be digested by the microbes that reside there, thus they can also be called microbiome-accessible carbohydrates (MACs) and offers the main energy for colonic microbes (41). These non-digestible carbohydrates have a huge impact on the gut microbiome, while dietary fiber is one of them that has been most clearly studied. As early as 45 years ago, Trowell's study showed that the cause of type 2 diabetes may be due to the lack of dietary fiber in the diet, while rapid changes in dietary habits led to a large-scale epidemic of diabetes in the Pima Indians, who changed their traditional diet containing sufficient non-digestible carbohydrates to a modern diet with scarce fibers and cereal products (42). These non-digestible carbohydrates, including human milk oligosaccharides (HMO), serve as not only anti-sticking agents that prevent pathogen adhesion and promote immune maturation, but also providing guidance for the maturation of the gut microbiome, contributing to human health (43, 44). For this purpose, specific non-digestible carbohydrates (NDC) are being produced, such as galactooligosaccharides (GOS) and fructooligosaccharides (FOS), which are currently being added to infant formulas to help the healthy development of the gut microbiome in China (45).

Application of the state-of-art sequencing methods has updated our knowledge of the function of dietary fiber, demonstrating that it plays an important role in affecting the microbiome structure and function. For example, the gut microbiome generates short-chain fatty acids (SCFAs) essential for gut health (46–49). There was a significant correlation between SCFAs levels and microbial community composition. Fermentation of dietary fiber could reduce the pH in the colon (5.5 to 6.5), and slow down the growth of several gram-negative pathogens, such as *Salmonella* and *Escherichia coli* (50, 51), providing a clear clue to the mechanism of dietary fiber regulating gut microbiome. Based on this theory, many studies have clearly described the negative effects of dietary fiber deficiency on gut microbiome and host, and demonstrated the benefits of short-term or long-term dietary fiber supplementation, especially for people with obesity and metabolic diseases. For example, an insufficient supply of dietary fiber will make certain beneficial bacteria strains disappear (52). More seriously, in chronic or intermittent dietary fiber deficiency, intestinal microbes will digest mucus glycoprotein secreted by the host, and erode the colonic mucus barrier. Lack of dietary fiber, combined with fiber deficiency and mucus corroding microbes, gives the mucosal pathogen, *Citrobacter rodentium*, more opportunities to contact the epithelium and cause fatal colitis (53).

In contrast, extra dietary fiber supplements seem to be beneficial. Eating 25 to 38 g dietary fiber each day was confirmed to be closely related to a reduced risk of type 2 diabetes by 20–30% (54). Furthermore, dietary fibers from chicory inulin and sugar beet pectin can be employed to control the immune response to nosocomial infections caused by non-fermenting Gram-negative bacilli, such as *Sphingomonas paucimobilis* (55), confirming again the importance of carbohydrates in the diet. The opposite is a so-called “weight loss” diet that are high in protein and low in carbohydrate, which will increase the number of *Bacteroides* and reduce *Firmicutes*, and then lead to higher risk of colon disease (56).

## Fats

Fat is mainly digested in the upper part of the small intestine, where it will be hydrolyzed into glycerin and fatty acids by various enzymes and bile acid salts, and then works as the main source of calories, satisfying half of the energy needs of us (57, 58). The gut microbiome also interacts intensively with dietary fat. On the one hand, gut microbiome impacts the energy balance of host, playing an important role in the absorption and metabolism of dietary fat (58, 59). What's more interesting is that gut microbes can even determine the distribution of fat in the body, thus forming different body shapes (60). On the other hand, the high-fat diet can effectively alter diurnal patterns of gut microbiome structure and function, finally achieving a new balance (57), but a lot of times it's harmful. High-fat diets are associated with a reduction in intestinal bacterial diversity, changes in membrane integrity, inducing increased permeability and increased lipopolysaccharide translocation, changes in the immune system, and generation of low-intensity systemic inflammation. Mouse experiments demonstrated that a high-fat diet causes a significant increase in intestinal deoxycholic acid (DCA) that promotes liver cancer (61). Although there is no evidence that there is a corresponding phenomenon in the human body, it cannot be ignored that some potential negative effects must exist. Another study further confirmed that the animal-based diet significantly increased the activity of bacterial genes that encode microbial bile salt hydrolases (22), which are necessary for DCA production by intestinal microorganisms (62). Elevated DCA levels may in turn lead to microbial disorders in the animal-based diet, as this bile acid inhibits the growth of *Bacteroides* and *Firmicutes* members (63). A high-fat diet also promotes the growth of *B. wadsworthia* (22), while the production of *B. wadsworthia*, H_2_S, is thought to inflame intestinal tissue and then case the inflammatory bowel disease (64). The mechanism behind this has also been fully analyzed. Firstly, *Bifidobacterium*-containing clusters are positively correlated with long-term dairy as well as baseline saturated fat intake, supporting the potential association with milk-related saturated fat (64); secondly, the animal-based diet increases the concentrations of fecal bile acid significantly; thirdly, the relative abundance of microbial DNA or RNA encoding sulfite reductase in the animal-based diet is significantly increased (22). All these findings support the idea that the effect of the diet-related factors on gut microbiome may induce inflammatory bowel disease.

Different from what is generally believed, the harm of the high-fat diet is not only for the old who have a low metabolic level but also for the young. In a recent study, 217 young adults (18 to 35 years old; body mass index below 28 kg per m^2^; 48% men) were invited to a 6-month randomized controlled-feeding trial by taking three different diets (20, 30, and 40% fat energy), demonstrated that the high fat intake of healthy young people seems to be related to adverse changes in the gut microbiome, fecal metabolic profiles and plasma pro-inflammatory factors, while these taking a lower-fat diet showed an increased α-diversity, increased abundance of beneficial *Blautia* and *Faecalibacterium* (65). Intestinal *Blautia* is related with decreased death risk from graft-vs. host disease, and *Faecalibacterium prausnitzii* is an anti-inflammatory commensal bacterium (66–68). Besides, the high-fat diet of young mothers is also important for the composition of their children's gut microbiome, which indirectly indicates that excessive fat intake is also unhealthy for infants (69). Therefore, excessive fat intake should be avoided at any stage of life to minimize the risks associated with it.

Many cases highlighted the benefits of the low-fat diet. Hyuju et al. found that the low-fat/high-fiber diet can promote anastomotic healing by regulating the microbiome in mice (70). In short, compared with low-fat/high-fiber (SD)-fed mice, Western diet (WD)-fed mice have an increased risk of anastomotic leakage, an increase in the relative abundance of *Enterococcus* in the intestinal lumen. After the operation, the microbial community of SD-fed mice (rather than WD-fed mice) returned to its preoperative composition. When WD-fed mice were exposed to the SD diet for 2 days before antibiotics and surgery, anastomotic healing was also significantly improved (70). Such study should be repeated in humans, and if the same results exist, it will be very helpful to develop specific diets to help patients recover from trauma.

However, an extremely low-fat diet is not always good, and the most important thing is to find a balance. Evidence from an animal experiment showed that long-term low-fat diet inhibited cholecystokinin (CCK) satiation, changed the caecal metabolome, and then reduced caecal weight in rats (71), which indicates that the low-fat diet must be used with caution, even for those who want to lost weight, before determining all potential side effects of it.

## Proteins

Protein and corresponding metabolites (mainly amino acids) are vital to human body functions and are also the main source of nitrogen for gut microbes. Digestion of protein starts in the stomach, where the pepsinogen can non-specifically degrade a variety of water-soluble proteins into polypeptides, oligopeptides and a small number of amino acids (72). After entering the intestine, these primary digestion products can be further degraded by trypsin and chymotrypsin into small peptides or amino acid molecules that can be absorbed (73). Gut microbiome seems to be involved in all the above processes and play an important role in downstream absorption, metabolism, transformation, and even mediate the interaction between dietary protein and host immunity (74–76). Amino acids can be further metabolized into a variety of microbial metabolites, which participate in a lot of host functions related to health and diseases. At the same time, different sources, concentration and components of dietary protein also affect the composition, structure, and function of the gut microbiome (77). In response to changes in dietary protein, microbial metabolites (including SCFAs, ammonia, amines, hydrogen, sulfide and methane, and other gases related to colon cancer and inflammatory bowel disease) have undergone significant changes (78–80).

Dietary guidelines from popular science often suggest high protein intake, especially from animal sources with diverse and enough essential amino acids, to combat muscle atrophy, obesity, weakness, osteoporosis, surgical stress and death rate. However, a proper ratio of protein to carbohydrate, or even a relatively low protein diet, is more recommended because excessive protein promotes the growth of pathogenic microorganisms, inducing a high risk of metabolic-related diseases (81). Residual nitrogenous compounds not absorbed by the small intestine are transferred to the distal intestine and metabolized by microorganisms in this part. Protein intake affects the quantity and species of microbial metabolites, but some of them are toxic, such as hydrogen sulfide, ammonia and indole compounds, which have potentially negative effects on host health (82–84). Some bioactive substances participate in various physiological processes of the host (76). Besides, high concentrations of protein supplementation will lead to an increase in the number of potential pathogens, which is due to the destruction of the homeostasis of the intestinal micro-ecosystem and the reduction of the number of beneficial microorganisms. This observation highlights the interaction between the gut microbiome and host health. Dietary protein altered gut microbiome affects host metabolism by regulating intestinal barrier function, intestinal motility and immune system. More seriously, there is evidence that after a high-protein diet, individuals with or without impaired renal function may experience deterioration in renal function (85, 86).

## Micronutrients

Micronutrients, namely minerals and vitamins, mean nutrients that the human body needs less, but are necessary for maintaining survival, growth, development and health. It is well-known that the “western diet”, lacking in micronutrients, drives nearly all modern chronic conditions by encouraging gut dysbiosis, while micronutrients also play an important role in this process (87). For example, obesity is related to changes in hormones, especially bone regulating hormones, such as vitamin D (88–90). Mild chronic inflammation can lead to an increase of pro-inflammatory cytokines by activating multiple signaling pathways, finally leading to obesity (91), while vitamin D has been recognized as having anti-inflammatory effects on various immune cells, although it has not been confirmed in randomized controlled trials (92). Research by Guo et al. showed that vitamin D can stimulate the expression of cathelidin antimicrobial peptide (CAMP) gene, which is expressed through immune cells and epithelial cells to enhance barrier function (93), suggesting that vitamin D is antibacterial. Also, vitamin D modifies epithelial cells' integrity, immune responses especially for the innate immunity, and the diversity as well as the composition of the gut microbiome (94), and is expected to alleviate inflammatory bowel disease by regulating homeostasis in the gut (95). In one clinical trial in patients with inflammatory bowel disease, receiving 1,200 IU/day of Vitamin D for 1 year reduced the relapse rate from 29 to 13% when compared with the placebo group (96). Dietary habits may also affect the synthesis of some micronutrients by affecting the structure and function of the gut microbiome. In the cecum, liver and kidneys, the vitamin K was primarily derived from microbes and was decreased by 32 to 66% in mice treated by large doses of antibiotics compared to untreated animals, which in turn seriously affects bone development (97), suggesting the importance of metabolites produced by the healthy gut microbiome, including various vitamins, to the body.

Recent reports have also shown that gut microbes affect mineral metabolism of the host, involving calcium, iron, magnesium, selenium, copper, zinc, and silver (98–108). Generally, the gut microbiome regulates the absorption of minerals, such as iron and calcium (109–111), helping them achieve a good balance for health. In turn, these minerals also have a great effect on the gut microbiome. For example, iron is responsible for intestinal bacteria to extract energy from nutrients obtained by the host (112). Studies have shown that the normal gut microbiome can improve the utilization of dietary iron by translating ellagic acid (EA) into urolithin A (UA), which does not bind Fe^3+^ and still maintains the biological function in the presence of Fe^3+^. UA inhibits the production of reactive oxygen species (ROS), and its increased synthesis has a positive impact on the health by protecting the host from oxidative stress and inflammation (113). Besides, due to the excreted p-hydroxyphenyllactic acid, *Lactobacillus fermentum* residing in the gut exhibits iron-reducing activity, catalyzing Fe^3+^ to Fe^2+^ (101). Fe^2+^, unlike Fe^3+^, can be absorbed by the host's enterocytes (114). In other words, the intestinal microbiota optimizes the dietary non-heme iron conversion in the intestine not only by increasing the content of Fe^3+^, but also supporting the reduction of Fe^3+^ to Fe^2+^, thereby improving the utilization of iron (115). Similar to these, other minerals interact with the gut microbiome, and this good interaction is the key to maintaining health, but that doesn't mean everyone needs mineral supplements. The most appropriate dietary recommendations are self-monitoring and supplementation of minerals and vitamins when deficiencies are identified.

## Food Additives

Food additives refer to artificial or natural substances added to food to improve the color, aroma, taste, as well as for the needs of anti-corrosion and special processing, including anti-caking agent, defoamer, acidity regulator, antioxidant, leavening agent, colorant, bleaching agent, enzyme preparation, flavor enhancer, color-protecting agent, preservative, sweetener, thickener, and spice etc. (116). More and more evidence showed that these food additives can disrupt the homeostasis of the gut, thus promoting tissue injury inflammatory response. For example, mice treated with emulsifiers carboxymethyl cellulose and polysorbate 80 developed biological disorders with overgrowth of mucus-degrading bacteria, leading to colitis in animals lacking interleukin-10 involving in anti-inflammatory and cell regulatory or toll-like receptor 5 (a cell receptor targeting bacterial flagellin) (117). Similarly, enhanced endoplasmic reticulum stress will be induced by maltodextrin in intestinal goblet cells, thus promoting mucus release and improving the host's susceptibility to colitis (118–121). Besides, by inducing changes in the composition and function of intestinal microflora, non-caloric artificial sweeteners (NAS) can lead to the development of glucose intolerance, even though it is regarded as very safe due to their low caloric content (122). Moreover, maternal exposure to NAS impacts progeny's metabolism and microbiome, including general downregulation of liver detoxification mechanisms and significant alterations in bacterial metabolites, posing a threat to the infant's metabolism (123). The potential harm of other artificial sweeteners should also not be ignored. Both of Splenda and Neotame can cause intestinal disorders, especially in people with Crohn's Disease-Like Ileitis (124, 125). Some dietary microparticles, such as titanium dioxide which is used as a colorant and food whitening agent, can inhibit macrophage phagocytic activity and work as adjuvants with bacterial stimuli, leading to the complex disorder of immune responses (126–128). The antimicrobial agent used for antisepsis is also unsafe. It has been well recognized that they can induce anxiety by remodeling gut microbiome (129, 130).

Although there are strict restrictions on the use of food additives in many countries (131, 132), the formulation of those standards is rarely based on systematic and rigorous scientific experiments (133). It may be wise to minimize the intake of food additives until the correlation between the dose and hazard of food additives is fully understood.

## Cooking and Processing

Cooking and processing are essential parts of most foods before they are eaten. Although few studies measured the effects of them on the gut microbiome, some existing experimental results may provide us with a deeper understanding. Carmody et al. pointed out that raw or cooked plant feeds reshaped the gut microbiome of mice differently, and its impact was attributed to the improvement of starch digestibility and the degradation of plant-derived compounds, while changes in the gut microbiota regulated the host energy status and similar phenomenon can also be detected in humans (134). In another more detailed study, the effects of three different cooking methods on gut microbiome were compared and analyzed with five different foods. The results showed that, compared to milder treatments (boiling), intense cooking techniques (roasting and grilling) increased the abundance of beneficial bacteria, such as *Ruminococcus spp*. or *Bifidobacterium spp*. However, for some foods (bananas or bread), intense cooking can reduce the level of healthy bacteria (135). Also, eating red meat or processed meat is linked to an increased risk of colorectal cancer partly due to the interaction between gut microbiome and carcinogens (136, 137). On the other hand, cooking utensils can also cause the accumulation of harmful substances in the cooking and processing, especially the use of aluminum cookers. Thirty eight percentage of aluminum intake accumulates in intestinal mucosa and disturbs the normal regulation of intestinal permeability, intestinal flora and immunity (138, 139).

## Effects of Gut Microbiome on Human Body Function and Diseases

Generally, almost all factors in the diet are closely related to the homeostasis of the gut microbiome, while unhealthy habit will definitely cause the decline of body function and even lead to pathological changes. The data of the recent decade's explosion clearly described the relationship between the gut microbiome and health from multiple perspectives, and explain the mechanism of many specific intestinal microorganisms in the development of diseases (32, 140). Dysbiosis of gut microbiome is closely related with gastrointestinal diseases (141), bone (142), mental (143, 144), aging-related inflammation (145), cancer (146), cardiovascular diseases (147, 148), circulatory rhythms (149), metabolic diseases (150–152), etc. Beyond to this, the co-evolution between gut microbiome benefits human a lot (153–155), including fighting against pathogens, stimulating the immunity (156–158), maintaining the intestinal barrier integrity, and generating micronutrients (160).

It is well-acknowledged that the diversity of gut microbiome is positively correlated with health, and a good gut microbiome not only keeps us from getting sick but also makes our bodies work more smoothly. For example, the metabolites of the gut microbiome can regulate a variety of physiological activities and have a strong signal transduction function (159). Microbial-derived SCFAs (butyrate, propionate, and acetate), appear in specific amounts, and their proportions will vary according to age, diet, and disease (160). The formation of SCFA is the result of the complex interaction between diet and the gut microbiota in the environment of the intestinal cavity. The level of SCFAs is largely affected by the proportion of intestinal commensal bacteria, and its dysbiosis can lead to unbalanced SCFAs, which serve as the main communication medium between the gut microbiota and the immune system (161). SCFAs also promote epithelial metabolism and decreases intracellular O_2_, contributing to the stabilization of HIF-1 (a transcription factor) and epithelial barrier function (162). It should be noted that the gut microbiome produces many other kinds of metabolites, such as bile acids and amino acid derivatives, which may also have important signaling functions (163). For example, gut microbiome-related bile acid metabolism regulates liver cancer via natural killer T cells (164, 165), suggesting these metabolic pathways associated with gut microbes may become an important target of precision medicine. However, in some abnormal intestinal conditions, the rapid increase of microbes-deprived metabolites may cause many diseases (166, 167). Unfortunately, the existing technology and analysis methods are still difficult to accurately locate the strains that play a key role. Therefore, more studies are needed to explore the core interactions between disease and the gut microbiome. But until all the mechanisms have been elucidated, it seems that we can enjoy the benefits of regulating the gut microbiome early, through a potentially harmless approach–dietary intervention.

## Dietary Intervention in Diseases

Recent studies have demonstrated that dietary intervention can significantly regulate the structure and function of the gut microbiome, and contribute to the health of gut microbiome and its host. A dietary intervention (calorie restriction) in obesity improved the abundance of *Akkermansia muciniphila* and then promoted metabolic health, helping to achieve good weight loss (168). Dietary fiber (mainly fructans and galacto-oligosaccharides) intervention increases the fecal abundance of *Bifidobacterium* and *Lactobacillus spp*, suggesting the possibility of precisely regulating certain microorganisms through specific dietary formulas (169). Besides, dietary intervention using functional foods decreased metabolic endotoxaemia and reduced biochemical abnormalities by improving gut microbiome in people suffering from type 2 diabetes, demonstrating that a high-fiber, polyphenol-enriched, and vegetable-protein-based diet may work as a potential therapy for the improvement of glycaemic control, dyslipidaemia, and inflammation (170, 171). Also, anti-inflammatory diets may reduce neuroinflammation through several indirect immune pathways from the gut microbiome and systemic circulation, introducing a new way to control Alzheimer's disease (AD) and neurodegeneration (172). All these data tell us that reasonable diet intervention may be an effective means to alleviate diseases or maintain health. The research of microbiome can not only help us to determine the microbes and dietary patterns that are directly beneficial to the body but also can be used as an indicator to predict the progress of disease and help us carry out the effective intervention in time. In the process of tackling the obesity pandemic, a lot of efforts have been devoted to formulating effective weight loss strategies. However, many dieters have failed to maintain weight loss for a long time and instead experienced excessive weight recovery cycles. The mechanism leading to the relapse of obesity after dieting remains largely elusive. Thaiss et al. developed a machine learning algorithm that can make personalized microbiome-based predictions on the degree of weight recovery after dieting, and found that the microbiome helps reduce flavonoid levels and energy consumption. And proved that flavonoid-based “post-biological” interventions have improved the secondary excessive weight gain (173). In other words, real-time and accurate tracking of microbiome dynamic changes is also necessary for timely and effective dietary intervention.

More than that, more and more studies show that a more appropriate diet can effectively improve physical function and even significantly reduce all-cause mortality. Recent evidence in mouse models shows that physical and emotional stress during exercise is highly correlated with changes in the microbial composition of the gastrointestinal tract. For example, induced exercise stress reduced *Turicibacter spp*. in the cecum but increased *Ruminococcus gnavus*, which has a clear role in intestinal mucus degradation and immune function (1, 174), providing an effective reference for athletes to solve fatigue, mood disturbances, underperformance and gastrointestinal distress. More importantly, a reasonable diet plan can help athletes shape gut microbiome which is more conducive to lactic acid degradation, which can effectively improve their exercise performance and achieve better results (175, 176). Similarly, dietary intervention is also suitable for ordinary people to improve their health and significantly reduce the incidence rate of many diseases (177, 178), so gut microbes can really tell you what to eat.

## Conclusion

Factors in the diet, including different dietary components (carbohydrates, fats, proteins, minerals, vitamins, etc.), food additives, cooking and processing, can change the structure and function of the gut microbiome, and these changes are closely related to maintaining the health of the body. Long term unhealthy eating habits, such as western diet, are an important factor in a variety of non-communicable diseases. Many years of research has depicted the basic principles of the interaction between diet and gut microbiome, while the dietary intervention program based on this has been proved to be effective. However, it cannot be ignored that many factors outside the diet that will also affect the composition of the gut microbiome, including age, genetics, smoking, sports activities and the like, so it is very challenging to accurately determine the specific role of diet in diseases. Also, it is difficult to draw definite conclusions on the therapeutic benefits of diet intervention for chronic diseases. As our review shows, most studies have been conducted on animals, and only a few human intervention studies exist. More randomized controlled studies are needed to ensure that enough subjects participate in the trial to fully understand the relationship between diet, gut microbiome and health.

## Author Contributions

QS draft the manuscript. QL provided critical comments on the manuscript.

## Conflict of Interest

The authors declare that the research was conducted in the absence of any commercial or financial relationships that could be construed as a potential conflict of interest.
